# Construction and Performance of the Hospice Care Index Claims-Based Quality Measure

**DOI:** 10.1093/geroni/igab046.239

**Published:** 2021-12-17

**Authors:** Michael Plotzke, Thomas Christian, Kim Groover, Zinnia Harrison, Ihsan Abdur-Rahman, Cindy Massuda

**Affiliations:** 1 Abt Associates, Saint Louis, Missouri, United States; 2 Abt Associates, Cambridge, Massachusetts, United States; 3 Abt Associates, Atlanta, Georgia, United States; 4 Abt Associates, Rockville, Maryland, United States; 5 CMS, Baltimore, Maryland, United States

## Abstract

As part of the Medicare Hospice Benefit (MHB), hospices submit claims containing information that allows policy makers to assess hospice quality, help policy makers improve the MHB, and increase patients’ experiences of care. We examine ten different hospice quality indicators related to the provision of services and patterns of live discharge. We calculated indicators using 100% Medicare fee-for-service (FFS) claims from October 1, 2018 through September 30, 2019. A hospice’s total score among all ten indicators is referred to as their Hospice Care Index (HCI), with a possible high score of 10. We examined all hospices with at least 20 discharges. After exclusion, we examined 4,155 hospices representing 1,562,003 beneficiaries. Most hospices earn a high HCI score: over 85% of hospices had scores of eight or more. At the same time, there were some lower scoring hospices: one in ten hospices scored seven on the index, and the remaining 4.9% scored six or lower. We find that on average hospices with higher HCI scores have better Consumer Assessment of Healthcare Providers and Systems (CAHPS®) Hospice ratings. Among hospices with a score of ten, 85.1% of caregivers reported they would definitely recommend the hospice vs. 82.9% of caregivers of patients receiving treatment from hospices with a score of seven or less. Using the HCI, the Centers for Medicare and Medicaid Services and hospice patient caregivers can assess hospices across a broad set of indicators. Policymakers and hospices should monitor these ten indicators to understand their performance relative to peers.

